# Nitrogen (N) Deposition Impacts Seedling Growth of *Pinus massoniana* via N:P Ratio Effects and the Modulation of Adaptive Responses to Low P (Phosphorus)

**DOI:** 10.1371/journal.pone.0079229

**Published:** 2013-10-21

**Authors:** Yi Zhang, Zhichun Zhou, Qing Yang

**Affiliations:** Research Institute of Subtropical Forestry, Chinese Academy of Forestry, Fu Yang, Zhe Jiang, P. R. China; Lakehead University, Canada

## Abstract

**Background:**

In forest ecosystems with phosphorus (P) deficiency, the impact of atmospheric nitrogen (N) deposition on nutritional traits related to P uptake and P use potentially determines plant growth and vegetation productivity.

**Methodology/Principal Findings:**

Two N deposition simulations were combined with three soil P conditions (homogeneous P deficiency with evenly low P; heterogeneous P deficiency with low subsoil P and high topsoil P; high P) using four full-sib families of Masson pine (*Pinus massoniana*). Under homogeneous P deficiency, N had a low effect on growth due to higher N:P ratios, whereas N-sensitive genotypes had lower N:P ratios and greater N sensitivity. The N effect increased under higher P conditions due to increased P concentration and balanced N:P ratios. An N:P threshold of 12.0–15.0 was detected, and growth was increased by N with an N:P ratio ≤ 12.0 and increased by P with an N:P ratio ≥ 15.0. Under homogeneous P deficiency, increased P use efficiency by N deposition improved growth. Under heterogeneous P deficiency, a greater P deficiency under N deposition due to increased N:P ratios induced greater adaptive responses to low P (root acid phosphatase secretion and topsoil root proliferation) and improved P acquisition and growth.

**Conclusions/Significance:**

N deposition diversely affected seedling growth across different P conditions and genotypes via N:P ratio effects and the modulation of adaptive responses to low P. The positive impact of N on growth was genotype-specific and increased by soil P addition due to balanced N:P ratios. These results indicate the significance of breeding N-sensitive tree genotypes and improving forest soil P status to compensate for increasing N deposition.

## Introduction

Low phosphorus (P) availability is a limiting factor of plant growth in many terrestrial ecosystems [1–3]. Plants have evolved a variety of adaptive responses to cope with low P availability, including modified root architecture, mutualistic symbiosis with mycorrhiza, increased exudation of root active compounds, and increased P use efficiency (PUE) [1,3,4]. Furthermore, changes in root diameter also play a key role since they influence root surface area and volume [4–6]. There is substantial variation in the extent of the adaptive changes and in P efficiency among different genotypes [3,4,7–9]. Therefore, an effective strategy for reducing the use of P fertilizers involves selecting and breeding plant cultivars with high P efficiency and favorable yields when P availability is limited [4,7–9]. 

In recent decades, with the rapid expansion of industry and intensive agriculture, increased atmospheric nitrogen (N) deposition has largely raised the availability of N relative to P. Increased N availability has become an important factor influencing soil nutrient status and vegetation productivity in terrestrial ecosystems [10–13]. The functional effect of N on root development and plant growth has been widely reported [12,14,15]. Moreover, dramatically increased N levels elevate an ecosystem’s N:P ratios [10,12,13], which might increase the degree of relative soil P deficiency. These factors potentially influence plant growth and P nutritional traits [10,12,14,15]. 

Compared with the nutrient conditions of agricultural soils, those of forest soils are generally harsher and more complex due to a lower extent of cultivation and fertilization [6,16–19]. In a forest site with poor nutrient status – typically those that are older or contain coniferous trees and have a very slow return rate of P from the litter to the soil surface – homogeneous P deficiencies impacting both the topsoil and the subsoil may occur [20–22]. In comparison, a heterogeneous P deficiency in which P availability is sufficient in the topsoil and declines substantially with soil depth might occur in forest sites with favorable nutrient status [23,24]. When elucidating how N deposition impacts plant growth and P efficiency under low P stress, both heterogeneous and homogeneous P deficiencies must be considered. 

The mechanisms underlying the N-P interaction in plants have been studied in recent years [10,14,25–27]. The biochemically dependent co-limitation hypothesis suggests that the acquisition of one nutrient depends on the availability of another [25–27]. Moreover, the use efficiency of one nutrient depends on the availability of another nutrient [25,26]. Studies in several grass species showed that increased N stimulates phosphatase activity via N:P stoichiometry effects, which potentially increases plant P uptake in a species-specific manner [14]. However, in forest ecosystems in which P conditions are harsh and complex, the impact of N deposition on nutritional traits related to P uptake and use have seldom been investigated.

The southern part of China is an important wood production area. Since highly weathered and acidified red soil dominates in this area, low P availability has seriously limited conifer wood plantation productivity [22,24,28]. Concurrently, the average inorganic N wet deposition in the southern part of China has increased in recent years, reaching 20–30 kg N ha^−1^ year^−1^ [12,13,15]. As a result, forest vegetation is encountering coupled conditions of P deficiency and N excess [12,24,29,30]. 

Masson pine (*Pinus massoniana*) is an important and native evergreen conifer in the southern part of China with an estimated plantation area of 5.7 million hectares [24,31,32]. Adaptive changes to low P stress in the root architecture as well as the root exudation of acid phosphatase have been demonstrated [24,31,32]. Here we conducted greenhouse experiments to characterize the impact of simulated N deposition on growth and P nutritional traits of Masson pine. We aimed to answer the following four questions:

(1) Will N deposition significantly impact growth and P nutritional traits of Masson pine under low P stress (2)? How are adaptive changes related to P uptake and use under P deficiency conditions, such as root architecture adaptations and the exudation of acid phosphatase, affected by N deposition (3)? Is the impact of N deposition on growth the same or different under heterogeneous and homogeneous P deficiency conditions (4)? Is the impact of N deposition on growth and P nutritional traits the same or different among Masson pine genotypes?

## Materials and Methods

### Ethical statement

All of the observational and field studies described herein were undertaken with relevant permission from the Laoshan Forest Farm, Chunan county, Zhejiang province, China.

### Experimental design and plant materials

The greenhouse experiments were performed during the 2011 growing season. The experiment was located in the Laoshan Forest Farm, approximately 32 km southwest of Hangzhou in the Zhejiang province (120.2 °E, 30.3°N, 150 m a.s.l.). The mean annual precipitation of the Laoshan Forest Farm is 1350 mm, while the mean annual temperature is 16.7°C. 

A 3 × 2 × 4 factorial design was adopted. Three P conditions and two N conditions were set up. Four full-sib families with varying growth performance were used as materials. They were selected from a group of full-sib families which were generated in a second-generation breeding garden in the Laoshan Forest Farm. The experiment comprised a total of 240 seedlings (3 P treatments × 2 N treatments × 4 families × 10 blocks). There was one seedling per family × nutrient treatments per block, within which the families were randomized. The experiment was set up in a semi-open greenhouse (30 m × 10 m) in the Laoshan Forest Farm in which a transparent plastic roof was used to keep off the rainwater and no walls were used to ensure that the environmental conditions inside the greenhouse including light intensity, temperature, and humidity were similar to those of the external environment. Columniform pots (35 cm deep, 20 cm in diameter) were used in the greenhouse experiment. The raw acrisol (one of the 30 major soil groups of the World Reference Base for Soil Resources, FAO, 2006) in the deep soil layer (at the depth of 1.0 m) of a forest stand in the Laoshan Forest Farm was dug up and used as a substrate. The substrate contained 1.42 mg kg^-1^ available P, 43.0 mg kg^-1^ hydrolyzed N, 37.6 mg kg^-1^ available K, and 7.1 g kg^-1^ organic matter (overall pH 5.05). The seedlings in each pot were irrigated with distilled water, and the water content of the soil was maintained at 20–40 kPa using a tensiometer. 

### N and P nutrient conditions

A high N and a low N treatment were set up. Each pot subjected to the high N treatment received experimental additions of N in the form of an NH_4_NO_3_ solution that was applied once a month from April to October. The total amount of the N addition was 0.4 g N m^-2^ month^-1^ and 2.8 g N m^-2^ year^-1^, which is equal to inorganic N wet deposition at 28 kg N ha^-1^ year^-1^. This level of N deposition and this ratio of NH^4+^ to NO^3−^ (1:1) were the same as the average level of atmospheric N wet deposition in the southern China regions with fast economic development [12,13]. For the low N treatment, no N was added to the substrate, and the N level in the substrate was kept at 43.0 mg kg^-1^ hydrolyzed N. 

The three P treatments included: (1) a homogeneous P deficiency condition in which P availability was evenly low in all soil layers, designated the “low-P (LP)” treatment group; (2) a heterogeneous P deficiency condition in which the P content was high in the top soil layer, lower in the middle layer, and lowest in the bottom layer, designated the “medium-low-P (MLP)” treatment group; and (3) a high P condition with evenly high P content in all layers, designated as the “high-P (HP)” treatment group. The depth of each soil layers was 10 cm. In the low-P (LP) treatment group, the three layers of soil contained no additional Ca(PO_4_)_2_, and were orderly put into pots to create a homogeneous P deficiency condition. In the medium-low-P (MLP) treatment group, the bottom-layer soil had no Ca(PO_4_)_2_, the middle-layer soil contained Ca(PO_4_)_2_ at a level of 0.05 g kg^-1^ soil, and the top-layer soil contained Ca(PO_4_)_2_ at a level of 0.2 g kg^-1^ soil. All three layers of soil were orderly put into pots, creating a heterogeneous P deficiency condition. In the high P (HP) group, three layers of soil contained Ca(PO_4_)_2_ at a level of 0.2 g kg^-1^ soil and were orderly put into pots.

### Seedling culture, harvesting and measurement

The experiment was started in March of 2011. Seeds of each Masson pine family were sown in each pot, and the seedlings were grown until harvest time in mid-November 2011. The following traits were measured or calculated as indicators of P efficiency: plant height, stem diameter, dry weight (DW) of each organ and of the whole seedling, P acquisition efficiency (PAE) and P use efficiency (PUE). The three layers of soil together with their roots were separated by a specific knife. The roots in each of the three soil layers were washed carefully to separate them from the soil. The root length (RL) was analyzed using a Win/MacRHIZO root analysis system (Régent Instruments, Quebec, Canada). The proportion of topsoil RL was calculated as the RL in the topsoil divided by that of the entire root system. All harvested roots and above-ground parts were dried and weighed. The dried plant organs were used to measure the P concentration, which was determined colorimetrically as phosphomolybdate. PAE and PUE were also calculated. PAE was calculated as the seedling P concentration multiplied by the seedling DW. PUE was calculated as the seedling DW divided by the seedling P content. 

For the measurement of acid phosphatase activity, the intact root system of each seedling was washed in distilled water and placed into 50 mL incubation medium with a substrate mixture including 6 mM p-nitrophenyl phosphate (pNPP), 1 mM dithiothreitol and 50 mM sodium acetate [Na-acetate] buffer at pH 5.0. The mixture was incubated at 25°C for 2 h. Subsequently, 200 μL aliquots of the reaction medium were collected and added to 200 μL of 4 M NaOH (to stop the reaction). The absorbances were read at 410 nm (Cecil CE 2501). The enzyme activity was expressed as acid phosphatase activity per unit root (Pase_root_, mg *p*NPP min^-1^ g^-1^ root fresh weight) and total root phosphatase activity (Pase_tot_, mg *p*NPP min^-1^, calculated as Pase_root_ × root fresh weight).

### Data analysis

All data sets were tested for normality using the UNIVARIATE procedure in SAS software (SAS Institute, Cary, NC, USA; 1996). All measured traits were analyzed by four-way (P treatments × N treatments × families × blocks) analysis of variance (ANOVA) and three-way (N treatments × families × blocks) ANOVA using SAS software. Trait variations between nutrient conditions and trait variations between families were analyzed using one-way ANOVA (SAS software). The means were compared using least significant differences (LSD). Linear correlations between seedling DW and PAE, PUE, root traits all were assessed by the SAS CORR procedure. Regressions and curve fittings were performed with the Statistical Analysis Toolpak of Microsoft Excel software. 

## Results

### Effects of P on growth and P nutritional traits

**Figure 1:**
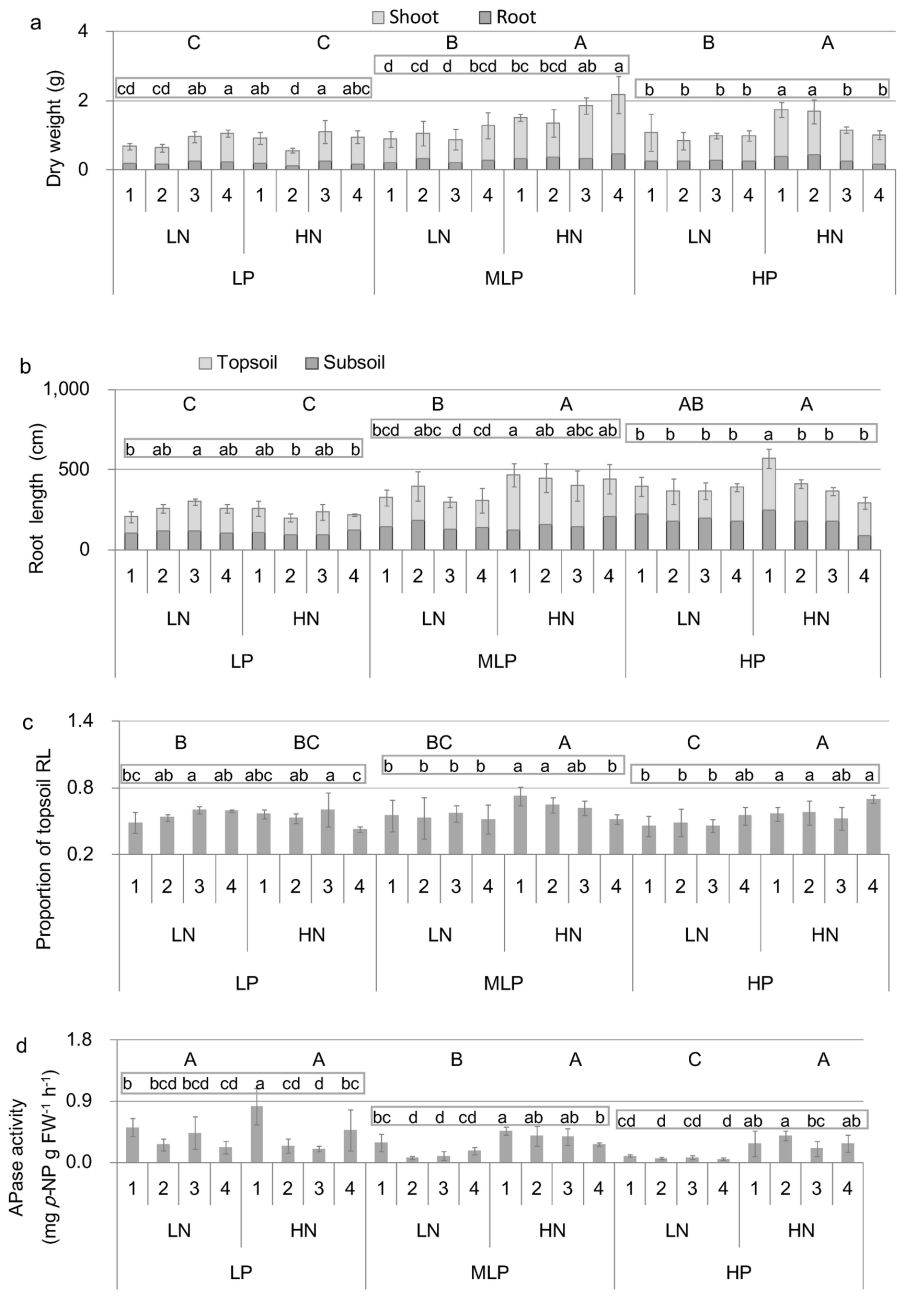
Major growth traits of each nutrient treatment. (a) Plant dry weight; (b) root length; (c) proportion of topsoil root length; (d) APase activity. The proportion of topsoil root length was calculated as root length in the topsoil divided by that of the whole root system. LP, low P (homogeneous P deficiency); MLP, medium low P (heterogeneous P deficiency); HP, high P; LN, low N; HN, high N; PAE, P acquisition efficiency; PUE, P use efficiency; APase, acid phosphatase. Error bars indicate the standard error of the mean. Significantly different (one-way analysis of variance [ANOVA], *p* < 0.05) average values between nutrient treatments are indicated by different capital letters, whereas significantly different (one-way ANOVA, *p* < 0.05) values between families within each P treatment are indicated by different lowercase letters.

Four-way ANOVA showed significant effects of P treatment on plant height, stem diameter, plant DW, PAE, and RL. No significant P effect on root to shoot ratios (R/S) and PUE was detected (Table 1). Seedling DW (Figure 1a), RL (Figure 1b), and PAE (Table 1) were all lower under the homogeneous P deficiency (LP) condition than that under the heterogeneous P deficiency (MLP) condition. No significant differences in major growth traits were detected between the MLP and HP conditions (Figure 1a, 1b, 1c). The activity of the root secreted acid phosphatase was much greater in the LP condition than that in the MLP and HP conditions (Figure 1d). 

**Table 1:** F values of four-way ANOVA analysis of major traits.

Factor	Height	Stem diameter	Dry weight	Root length	R/S	PAE	PUE
P	5.95**	26.84**	14.90**	31.13**	0.33	24.93**	2.41
N	3.55	38.04**	24.25**	4.12*	29.58**	0.43	9.44**
P × N	1.60	5.30**	5.67**	4.66*	0.19	0.91	0.48
Genotype	9.73**	2.13	1.33	1.72	9.33**	5.13**	1.92
Blocks	1.64	0.49	0.05	0.05	0.18	0.41	0.44

PAE, P acquisition efficiency; PUE, P use efficiency. Significance of F values of analysis of variance (ANOVA) is denoted by * 0.01 < *p* < 0.05, **p < 0.01.

### Effects of N on growth and P nutritional traits

Significant effects of N and the N × P interaction on seedling DW and RL (Table 1) were found, indicating that N deposition affect Masson pine diversely across different P conditions. Under the LP condition, there was no significant N effect on seedling DW and RL. However, a significant N × genotype interaction effect was detected, and N affected growth in a genotype-specific way. One of the tested families (family 1) showed high sensitivity to N deposition and exhibited increased DW and PUE under high N conditions. 

The degree of the N effect was much greater in the MLP and HP treatments than in the LP condition. Under the MLP condition, three tested families (families 1, 3 and 4) were highly sensitive to N addition, and exhibited significant growth improvement after N addition (Figure 1a; Table 2). Under the MLP condition, the PUE of families 1 and 4, and the PAE of families 2 and 3 were increased by simulated N deposition (Table 2). RL was also significantly increased by N deposition under the MLP condition but not under the LP condition (Figure 1b). The relative allocation of RL in the topsoil layer was increased by simulated N deposition under the MLP and HP conditions (Figure 1c). Root-secreted acid phosphatase activity was not affected by N addition under the LP condition, whereas it was significantly increased by N addition under the MLP condition (Figure 1d). 

**Table 2:** Trait average and F values of three-way ANOVA of major traits under LP.

Family code			LP					MLP			
			DW (g)	N/P ratio	PAE (mg)	PUE (g mg^-1^)		DW (g)	N/P ratio	PAE (mg)	PUE (g mg^-1^)
1	LN		0.68 B a	11.8 B a	0.94 A b	0.73 B a		0.89 B a	8.7 B b	1.52 A a	0.59 B a
HN			0.92A b	39.2 A a	0.49 B b	1.93 A a		1.51 A a	26.5 A b	1.17 A a	1.46 A a
2	LN		0.64 A b	15.0 A a	0.77 A b	0.94 A a		1.06 A a	8.3 A b	1.37 B a	0.85 A a
HN			0.57 A b	17.9 A a	0.59 A b	1.06 A a		1.36 A a	10.5 A b	2.17 A a	0.63 A b
3	LN		0.96 A a	13.8 B a	0.97 A a	1.02 B a		0.89 B a	10.9 B a	0.88 B a	1.05 A a
HN			1.10 A b	26.0 A a	0.82 A b	1.37 A a		1.86 A a	21.2 A a	1.57 A a	1.32 A a
4	LN		1.06 A b	31.3 A a	0.68 A b	1.84 A a		1.29 B a	12.0 B a	1.41 A a	0.98 B b
HN			0.95 A b	25.0 A a	0.72 A b	1.35 A a		2.18 A a	21.2 A a	1.55 A a	1.53 A a
F value	N		0.48	3.45*	4.36*	3.42*		20.43**	46.84**	4.60*	4.13*
	G		7.77**	8.14**	1.27	2.50		2.61	5.46**	2.48	1.61
	N×G		3.36*	3.77*	1.24	4.88*		0.97	2.76	3.16*	1.65
	Blocks		1.95	0.94	1.11	0.47		0.62	0.79	0.19	0.81

LP, low P (homogeneous P deficiency); MLP, medium-low-P (heterogeneous P deficiency); HP, high P; LN, low N; HN, high N; PAE, P acquisition efficiency; PUE, P use efficiency; DW, dry weight; G genotypes. Significance of F values of analysis of variance (ANOVA) is denoted by * 0.01 < *p* < 0.05, **p < 0.01. Trait averages with different capital letters are statistically different between LN and H N, whereas trait averages with different lowercase letters are statistically different between LP and MLP at the 5% level according to one-way ANOVA.

### The role of N:P ratios

The N:P ratios were 11.8–31.3, 17.9–39.2, 8.7–12.0, and 10.5–26.5 under the LP-LN, LP-HN, MLP-LN, and MLP-HN conditions, respectively (Table 2). In general, N:P ratio (ratio of seedling N concentration to P concentration) was largely increased by N addition and decreased by P addition. Moreover, large differences in N:P ratios were detected among families (Table 2). Under low N conditions, N:P ratio was found to be related to a growth response to N addition. With N:P ratios ≤ 12.0 (family 1 in LP-LN, all families in MLP-LN), seedling growth was largely increased by N addition (Table 2). In contrast, when N:P ratios ≥14.0 (families 2, 3, 4 in LP-LN), seedling growth was not sensitive to N addition but was increased by P addition (Table 2). 

### Correlation between growth and P nutritional traits in response to N conditions

**Figure 2:**
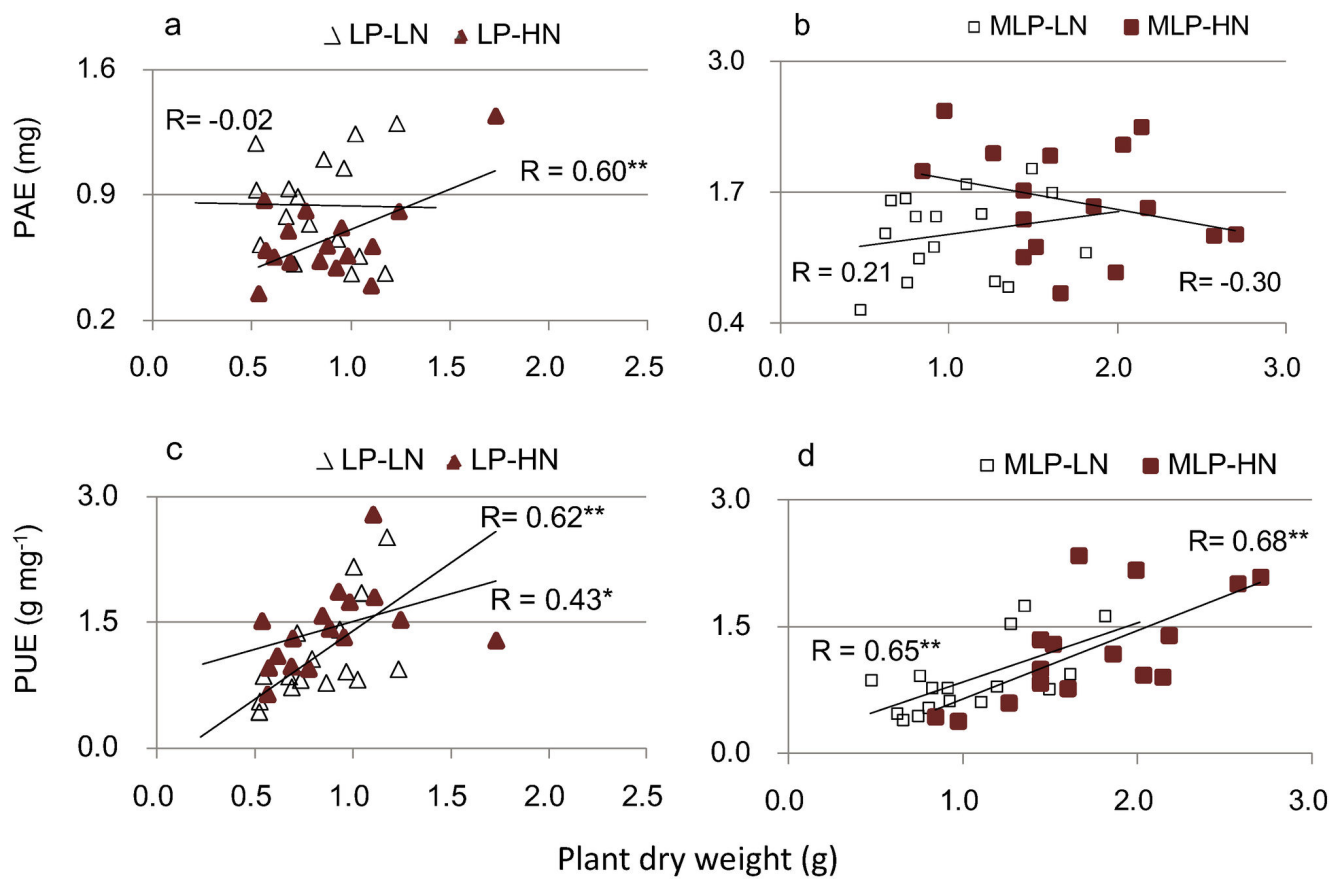
Linear correlations between plant dry weight and PAE, PUE. (a) (b) correlation between dry weight and PAE; (c) (d) correlation between dry weight and PUE. PAE: P acquisition efficiency; PUE: P use efficiency; LP: low P (homogeneous P deficiency); MLP: medium low P (heterogeneous P deficiency); LN: low N; HN: high N. Level of significance of correlation is denoted by: *0.01 < *p* < 0.05, **p < 0.01. PAE, P acquisition efficiency; PUE, P use efficiency.

**Figure 3:**
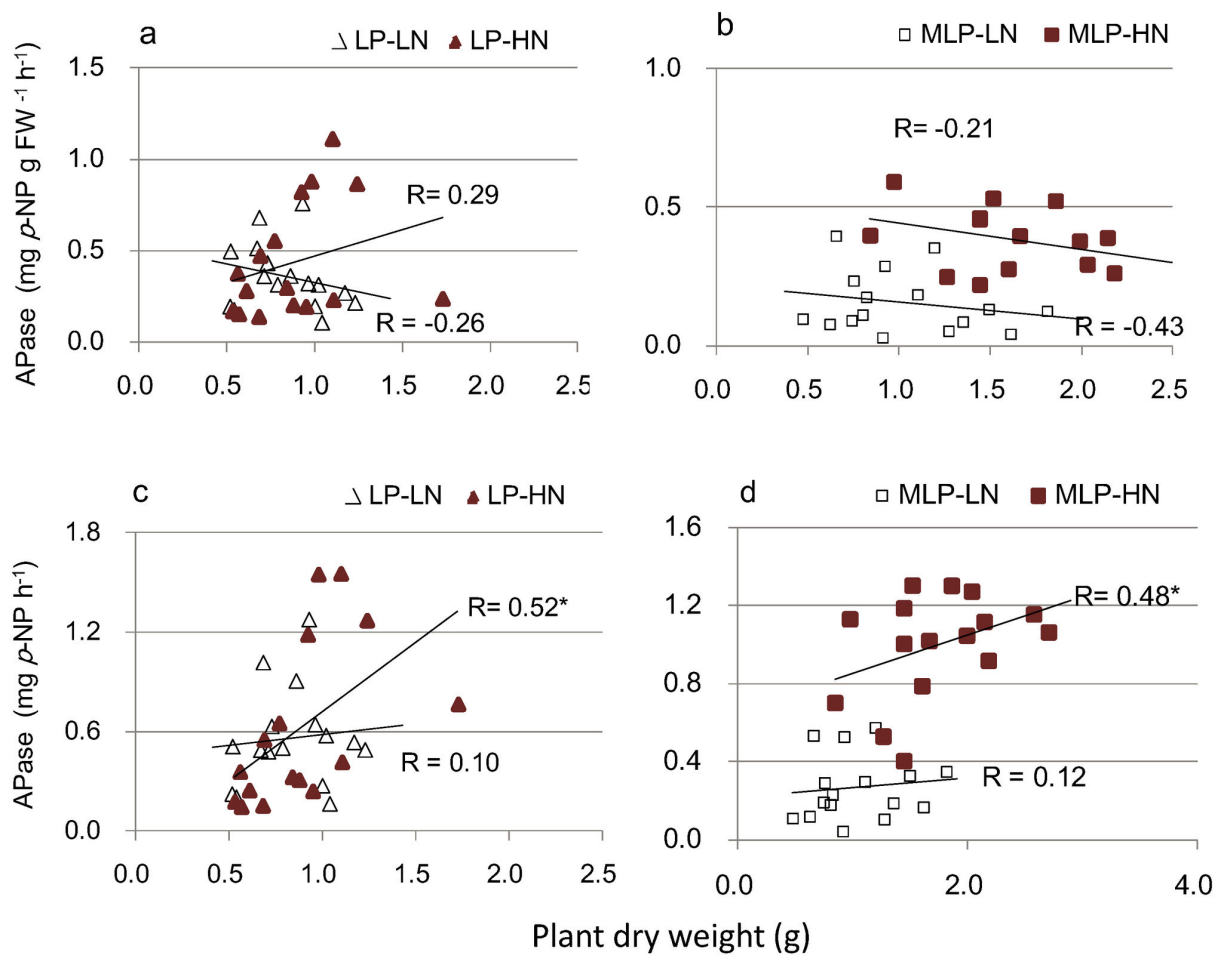
Linear correlations between plant dry weight and APase activity. (a) (b) correlation between dry weight and APase_root_; (c) (d) correlation between dry weight and APase_tot_. APase_root_, acid phosphatase activity per unit root; APase_tot_, total root acid phosphatase activity calculated as APase_root_ × root fresh weight; LP, low P (homogeneous P deficiency); MLP, medium low P (heterogeneous P deficiency); LN, low N; HN, high N. Level of significance of correlation is denoted by *0.01 < *p* < 0.05, **p < 0.01.

A significant positive correlation between seedling DW and PAE was observed within the LP-HN treatment group but not within the other nutrient conditions (Figure 2a, 2b). In comparison, there were positive correlations between plant DW and PUE within all nutrient conditions (Figure 2c, 2d). There was no significant correlation between seedling DW and acid phosphatase activity per unit root (Pase_root_) under each nutrient condition (Figure 3a, 3b). A significant correlation between seedling DW and total phosphatase activity (Pase_tot_, Pase_root_ × root mass) was detected under high N conditions (LP-HN and MLP-HN) but not under low N conditions (LP-LN and MLP-LN) (Figure 3c, 3d). 

## Discussion

### Genetic variation in P nutritional traits under varying nutrient conditions

Both homogeneous P deficiency (P is deficient in the entire soil profile) and heterogeneous P deficiency (P is deficient in the subsoil but sufficient in the topsoil) might occur in forest stands [20,24,28]. The present study demonstrated that homogeneous P deficiency (LP) largely suppressed plant growth under both low and high N conditions, whereas growth was improved by the partial addition of P (heterogeneous P deficiency, MLP). Substantial genotypic variation in major growth and P nutritional traits including plant DW, RL, relative allocation of the topsoil root, APase activity, PAE, and PUE were detected under both high N and low N conditions (Table 2; Figure 1). This finding indicated the potential for breeding genotypes with high P efficiency and improved biomass production under coupled conditions of P deficiency and N deposition. Although the promotion of P efficiency in plants can be achieved through improving PAE and/or PUE, the relative significance of PAE and PUE varies among plant species and environmental conditions [1,7,24,33]. The present study found a great contribution of PUE to growth under all nutrient conditions, whereas a large contribution of PAE was found only under LP-HN (homogeneous low P and low N) conditions (Figure 3). It has been suggested that when a P supply is extremely limited, PAE could be more important than PUE [33]. Our results support above findings since the largest degree of P deficiency and the highest plant N:P ratios were seen under the LP-HN condition. 

### N:P ratios and the effect of N deposition on growth

Increased atmospheric N deposition might dramatically influence plant root traits and growth performance [12,14,15]. A forest stand exposed to high N deposition responds with very markedly increased growth [12,14]. However, several studies have detected the rise in N deposition as a likely cause of forest decline [15,17]. In the present study, N deposition diversely impacted growth and P nutritional traits of Masson pine across P conditions. The positive impact of simulated N deposition on growth and P nutritional traits was much greater under heterogeneous P deficiency (MLP) and high P (HP) conditions than under the homogeneous P deficiency (LP) condition. 

Further analysis revealed that the varying growth response to N was related to the effects of the N:P ratios. The growth response of plants to N or P was suggested to be dependent on the relative availability of N and P, which was reflected by the N:P ratios in the plant tissues [10,25–27]. At the vegetation level, N:P ratios < 10 and > 20 often (but not always) correspond to N- and P-limited biomass production, whereas that of 10 < N:P<20 often implies co-limitation [10]. The growth rate is increased by the addition of either nutrient at the N:P threshold value, whereas it can be increased only by the least limited elements when the N:P ratio deviates from the threshold [10,25,26]. In our results, lower N:P ratios should be responsible for the large growth response to N addition under heterogeneous P deficiency. A likely N:P threshold of 12.0–14.0 was detected since the growth rate could be increased by the addition of N with an N:P ratio ≤ 12.0 (family 1 in LP-LN, and all families in MLP-LN), and could be increased mainly by the addition of P with an N:P ratio ≥ 14.0 (Table 2). The degree of N impact on growth was lower under the homogeneous P deficiency condition due to a low P concentration and a high N:P ratio, whereas it increased with increasing soil P availability via the effects of balanceable N:P ratios. Since the soil P availability was much greater in the conifer-broadleaf mixed forest than that in the pure conifer forest due to the faster return rate of P from the litter to the topsoil [19,34,35], the mixed forest type should be preferred for acquiring greater soil P availability and subsequently obtaining greater growth improvement by N deposition via a balanceable N:P ratio.

Under the homogeneous P deficiency (LP) condition, a significant effect of the N × genotype interaction was detected, and the degree of N effect was diverse across families (Table 2). Family 1 showed the largest sensitivity to N addition and exhibited increased growth under a high N condition. This was related to the effects of balanced N:P stoichiometry since family 1 had a lower N:P ratio (11.8) than did the other families under the LP-LN condition (Table 2). The intraspecific variation of the N:P ratio could be more important than the interspecific variation [29]. Taken together with those of earlier reports, our results confirm that the genotype-specific N:P ratios and growth response to N should be well regarded and used. In breeding genotypes with balanced N:P ratios and positive growth response to N addition, growth benefits could be obtained under increasing N deposition. 

### Mechanisms underlying the effect of N deposition on growth and P nutritional traits

According to the biochemically dependent co-limitation hypothesis, the acquisition and use of a single nutrient depends on the availability of another [10,14,25–27]. Therefore, nutrient acquisition and use efficiency are involved in the mechanisms that cause N–P interactions [25,26,36]. Under the homogeneous P deficiency condition in the present study, the PUE values of families 1 and 3 were increased by N deposition. In contrast, the PAE values of families 2 and 3 were largely increased by N deficiency under the heterogeneous P deficiency (MLP-HN) condition, presumably due to the elevated root acid phosphatase activity resulting from the N addition. These results corresponded with those of earlier reports and supported the biochemically dependent co-limitation hypothesis [25–27]. Moreover, our results demonstrated that the mechanisms underlying the N–P interaction varied across different nutrient conditions. 

Enhanced root acid phosphatase activity was a major adaptive response of plants to low P stress. Plants also enhance exploration and exploitation of enriched topsoil P by inducing adaptive changes in root architecture [24,31–33,37,38]. Increased N levels might stimulate phosphatase activity of several grass species via N:P stoichiometric effects, which potentially increases P uptake in a species-specific manner [14]. However, very little is known about the effect of N deposition and N:P ratio on the adaptive responses of tree species to heterogeneous P deficiency. In the present study, adaptive responses to heterogeneous P deficiency including root acid phosphatase secretion and root architecture adaptations were both enhanced by N deposition. We concluded that N deposition largely increased both the availability of N and the relative deficiency of P via the effect of the elevated N:P ratio. A higher N:P ratio and greater P deficiency under N deposition led to enlarged adaptive changes to low P levels including root acid phosphatase secretion and topsoil root proliferation, which subsequently led to enhanced P acquisition and promoted growth under the high N condition. Positive correlations between seedling DW and total root phosphatase activity were detected under high N (MLP-HN and LP-HN) conditions (Figure 3), further confirming the significance of N-stimulated root phosphatase activity for growth improvement due to high N availability. 

A number of previous studies demonstrated that progeny testing and assessment of growth and nutritional traits at juvenile age may be cost-effective and feasible. Significant correlations between juvenile (1-3 years) and mature (10-15 years) growth were detected in Sikta spruce (*Picea sitchensis*) [39], loblolly pine (*Pinus taeda* L.) [40] and rubber tree (*Hevea* *spp.*) [41]. Relationship between nutrient-related traits of seedlings and adult trees was detected in Douglas fir (*Pseudotsuga menziesii*) [42]. According to these reports, the present results drawn from Masson pine seedlings might provide moderate references for silviculture at both juvenile and older age. However, a systematic investigation of the juvenile-mature correlations of nutritional traits in Masson pine would be beneficial for scaling these results up to mature trees and applying the present results to a breeding program. Moreover, as most trees form mycorrhizal associations that enhance the acquisition of P by tree roots [43], investigating the effects of mycorrhizae is necessary for the thorough disclosure of how N deposition impacts plant growth and P efficiency under low P conditions. These two issues should be considered in the following studies.

## Conclusions

Increased PUE and PAE contributed to the growth improvement seen after simulated N deposition under homogeneous and heterogeneous P deficiencies, respectively. Under heterogeneous P deficiency, a larger degree of P deficiency under N deposition derived from increased N:P ratios induced greater adaptive responses to low P levels including root acid phosphatase secretion and topsoil root proliferation. This subsequently led to improved PAE and growth under N deposition. The degree of growth response to N increased with increasing soil P availability via the effects of balanceable N:P ratios, indicating the significance of improving soil P status to compensate for increasing N deposition. We also demonstrated genotype variations in growth responses to N deposition, indicating the significance of breeding N-sensitive conifer genotypes to cope with increasing N deposition and obtain growth benefits under P deficiency and N deposition conditions. 
